# Concurrent Angiomyolipoma and Clear Cell Renal Cell Carcinoma in the Same Kidney: A Rare Finding in a Patient without Tuberous Sclerosis

**DOI:** 10.1155/2021/6663369

**Published:** 2021-09-01

**Authors:** Amal Fekkar, Kaoutar Znati, Fouad Zouaidia, Hafsa Elouazzani, Zakia Bernoussi, Ahmed Jahid

**Affiliations:** Department of Pathology, Ibn Sina University Hospital Center, Rabat, Morocco

## Abstract

Synchronous renal cell carcinomas (RCC) and angiomyolipomas (AML) occurring in the same kidney are rare. Cases in the setting of tuberous sclerosis (TS) have been reported in the literature. However, the association of these tumors in the same kidney without TS is even more rare. We report here a case of a clear cell renal cell carcinoma (CCRCC) associated with an AML in the same kidney in a 42 years old female lacking the TS diagnostic criteria. The patient underwent a radical nephrectomy. Six months after surgery, the patient is healthy without signs of tumor recurrence or distant metastasis.

## 1. Introduction

AML are the most common benign tumor of the kidney, however, representing just 1% of surgically removed renal tumors [1]. It belongs to the family of perivascular epithelioid cell tumors (PEComas) and may occur sporadically or in patients with TS. The coexistence of RCC with AML in the same kidney is an uncommon finding, and nearly 80 cases with and without TS have been reported [2]. However, the majority of cases were in patients with TS [3, 4]. TS is an autosomal genetic disorder characterized by an increased risk of developing AML, renal cysts, and RCN. The clinical manifestations are broad, affecting not just the kidneys but also the brain, skin, lungs, and heart [5]. The clinical diagnosis of TS is based on the revised Gomez criteria. Rarely, RCN and AML can occur in the same kidney, sporadically, without TS, as synchronous or metachronous tumors.

## 2. Case Report

A 42-year-old female, who underwent 18 years ago, extracorporeal shockwave lithotripsy for right kidney stones, presented with six months history of right lumbar region pain associated with dysuria without hematuria. Physical exam showed right-sided abdominal tenderness.

**Figure 1:**
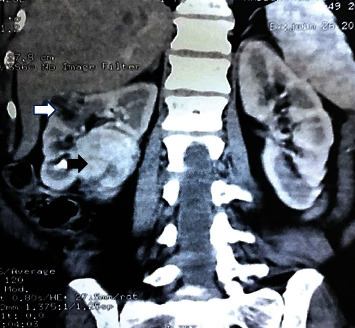
Computerized tomography urography scan showing two tumors in the midportion of the right kidney with distinct radiologic appearances: the external mass (white arrow) is a fat-containing tumor suggesting AML. The internal tumor (black arrow) is solid demonstrating a heterogeneous enhancement pattern.

Computed tomography (CT) urography (Figure 1) showed two masses in the midportion of the right kidney with distinct radiologic appearances. The external mass measuring 24 × 23 mm was a fat containing tumor suggesting AML. The internal tumor was fleshy and large measuring 49 × 40 mm. It deformed the renal contour and demonstrated a predominantly heterogeneous enhancement pattern. There was no evidence of lymph node metastases.

The other laboratory findings were within normal limits.

A right radical nephrectomy was done.

The patient had an uneventful postoperative recovery, and no neoadjuvant treatment has been administered. Six months after surgery, the patient is healthy without signs of tumor recurrence or distant metastasis.

**Figure 2:**
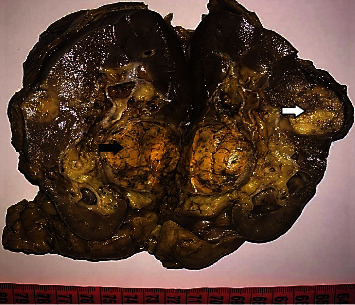
Gross examination revealing two different renal masses: the largest mass (black arrow) was encapsulated and firm composed of golden yellow soft areas with hemorrhagic changes. The smaller mass (white arrow) was well defined and had yellowish, glistening cut surface.

On gross examination (Figure 2), the kidney was slightly enlarged weighing 280 g and measuring 130 × 70 × 65 mm. The cut surface revealed 2 masses in the midportion. The largest mass of 47 × 40 mm was encapsulated and firm with a heterogeneous appearance, composed of golden yellow soft areas with hemorrhagic changes. The smaller mass measuring 25 × 20 mm was well defined and had yellowish, glistening cut surface. It was located at 3 cm from the first lesion.

**Figure 3:**
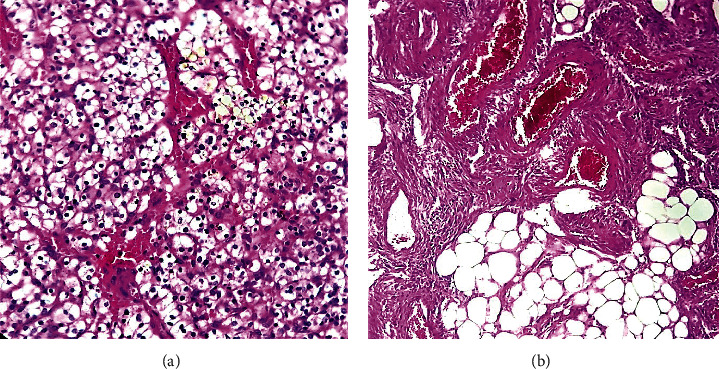
Hematoxylin and eosin staining demonstrating as follows. (a) CCRCC composed of nests of cells with clear cytoplasm, surrounded by a network of abundant thin-walled blood vessels. (b) AML composed of aggregates of thick-walled blood vessels, admixed with mature adipose tissue and smooth muscle cells.

Microscopic examination of the largest mass showed a malignant proliferation composed of cells with distinct cell membranes, optically clear cytoplasm, and rounded nuclei slightly larger than a red cell with inconspicuous nucleoli (consistent with Fuhrman grade 1). The cells were mainly arranged in sheets and compact nests surrounded by a regular network of thin-walled blood vessels (Figure 3). These features were consistent with the diagnosis of CCRCC.

The smaller lesion corresponded histologically to an AML composed of aggregates of thick-walled blood vessels, admixed with large mature fat cells and smooth muscle cells (Figure 3).

**Figure 4:**
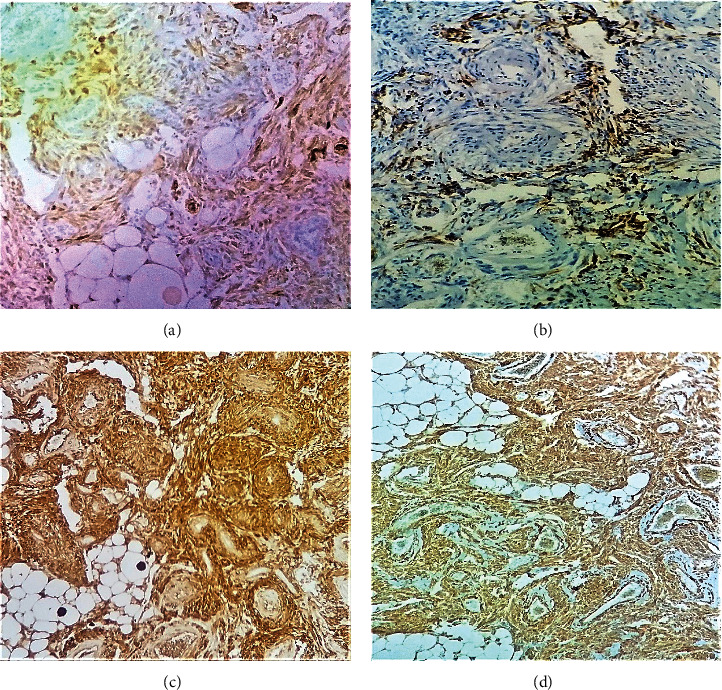
Immunohistochemical study of the AML showing the positivity of (a) Melan A, (b) HMB45, (c) H-Caldesmon, and (d) smooth muscle actin.

In the immunohistochemical study, AML showed positive immunostaining for melanocytic markers (HMB-45 and Melan-A) and for smooth muscle markers (smooth muscle actin and H-caldesmon) (Figure 4).

## 3. Discussion

The simultaneous occurrence of two different renal tumors in the same kidney is rare. The combination of these neoplasms can take different aspects. We distinguish separate tumors, collision tumors, and composite tumors. A collision tumor is a meeting of two tumors arising in independent topographical sites. A composite (hybrid) tumor consists of two different neoplasms associated in the same renal nodule. Separate tumors are simultaneous neoplasms of different types occurring in two distinct settings, in association with hereditary disorders or sporadically [3]. In our case, the tumors had a sporadic occurrence and were separated by normal renal tissue.

There have been rare reports of the simultaneous occurrence of RCC and a variety of benign and malignant renal neoplasms within the same kidney. AML with concomitant CCRCC is seen commonly in patients with TS and rarely in patients without this syndrome [3, 4].

In our case, we used the clinical criteria (the revised Gomez Criteria) to rule out the diagnosis of TS; therefore, the patient underwent various clinical and paraclinical examinations from several medical specialists notably in dermatology, cardiology, ophthalmology, neurology, radiology, and dental medicine, and no major or minor clinical feature of TS was determined. In the 2012 International Tuberous Sclerosis Complex Consensus Group, comprising 79 specialists from 14 countries, organized into 12 subcommittees, each led by a clinician with advanced expertise in TS complex and the relevant medical subspecialty, special attention was given to evaluate the sensitivity and specificity of clinical findings with respect to TS diagnosis, and they concluded that clinical features of TS complex continue to be a principal means of diagnosis. The identification of either a TSC1 or TSC2 pathogenic mutation in DNA from the normal tissue is sufficient to make a definite diagnosis of TS but a significant fraction 10% to 25% of TS patients has no mutation identified by conventional genetic testing, and a normal result does not exclude TS or has any effect on the use of clinical diagnostic criteria to diagnose TS [6] (Table 1).

TS is an autosomal dominant disorder caused by the pathogenic mutation of TSC1 or TSC2 genes. It is characterized by mental retardation, seizures, and the development of cellular proliferations involving various organs, such as AML, subependymal giant cell tumors, cutaneous angiofibromas, cardiac rhabdomyomas, lymphangioleiomyomatosis, and pulmonary multifocal micronodular hyperplasia. Renal pathology is encountered in about 60% of TS patients, including renal cysts, AML, and epithelial neoplasms such as RCC and oncocytomas [4].

CCRCC are the most common type of RCC accounting for 65-70% of all renal cancers [1]. They are mostly sporadic and typically solitary cortical tumors. Like other renal neoplasms, they could be found incidentally or manifested by nonspecific symptoms such as hematuria or lumbar pain. Radiologically, there is a large overlap of imaging features between benign and malignant renal masses that often makes difficult a correct characterization of these tumors [7]. CCRCC typically shows a heterogeneous consistency, demonstrates a high signal intensity on T2-weighted magnetic resonance imaging (MRI), and is hypervascular on dynamic contrast-enhanced CT or MRI examinations [8]. The diagnosis is made by pathological features. They are morphologically composed of cells with clear or eosinophilic cytoplasm arranged most commonly in solid alveolar and acinar pattern and contain a regular network of small thin-walled blood vessels. The tumor cells express epithelial markers, PAX8, and CD10. Carbonic anhydrase IX had characteristically a diffuse membranous overexpression in 75-100% of CCRCC. In most of these tumors, there is a deletion or an unbalanced chromosomal translocation (3;6, 3;8, or 3;11) that is due to either somatic mutations or hypermethylation-induced inactivation in patients harboring the VHL gene [9].

AML are the most common mesenchymal tumors of the kidney, known for their variable morphology and benign clinical course. They are part of the PEComas family. Most cases of AML are sporadic with higher incidence in women (1/4) and a mean age of 38 years. In patients with TS, AML occur in younger age, and they are usually small, bilateral, and multiple, whereas sporadic AML are usually unilateral and solitary [10]. The clinical features depend on the presence or not of TS. In TS, AML are usually asymptomatic, whereas sporadic AML are more likely larger manifested by flank pain, heamaturia, palpable mass, or revealed by a massive retroperitoneal hemorrhage due to rupture, representing the most dangerous complication. Radiologically, classic AML is the only benign solid renal mass that can be characterized with confidence by imaging through the identification of fat without calcifications on CT or MRI; however, approximately 5% of AMLs are “fat poor,” and there are rare cases of renal malignancies that contain fat, mimicking AML [7]. Morphologically, AML are composed of varying quantities of myoid spindle cells, adipose cells, and dysmorphic blood vessels. It is believed that these diverse tissue elements arise from the same cell of origin, the perivascular epithelioid cell, with its potential to differentiate into adipocytes, smooth muscle cells, endothelial cells, and HMB45 immunoreactive cells [11]. Immunohistochemically AML are characterized by the coexpression of melanocytic (HMB-45, Melan-A) and smooth muscle markers. Epithelial markers are always negative. There are several variants of renal AML, distinguished by histological features. The most important to recognize is the epithelioid AML, because of his malignant potential. It is also the subtype that raises the most the problem of differential diagnosis [12]. It can mimic a high-grade sarcomatoid renal cell carcinoma [9]. Histologically, the TS-associated AML are more likely to have an epithelioid component compared with the sporadic AML, which are typically triphasic [12, 13], like in our case.

Both sporadic and TS-associated AML frequently show a loss of heterozygosity at the TSC2-containing region on chromosome 16p and occasionally at the TSC1 region on chromosome 9p34 [9].

In a study, all patients with concomitant sporadic AML and RCN were female. The age range was 49 to 87 years with a mean age of 63.3 years [12]. The patient in our case was a 42-year-old female. Jimenez et al. studied 36 cases of coexistent RCN and AML and found that the majority of tumors associated with AML were CCRCC (62.5%), whereas the second most frequently associated tumor was oncocytoma [14]. Authors recommend the consideration of the possible coexistence of RCC with AML in cases with or without TS in the management of radiologically diagnosed AML accompanied by other renal masses of indeterminate nature [2].

The association of RCC and AML in the same kidney may be coincidental, or there could be a pathogenetic relationship [11]. The theory of cancer stem cells (CSCs) suggests that two or three dissimilar renal tumors could arise from CSCs. Another hypothesis explaining the pathogenesis of the coexistence of different renal tumors might be the evolution of one subtype to another [5]. Some studies claim that the RCN can develop from a preexistent AML [12].

The rarity of the reported cases and the limited follow-up data do not allow an accurate analysis of the prognosis of this combination of neoplasms; although, it seems that the prognosis depends mainly on the pathological stage of the RCC [14].

## 4. Conclusion

The coexistence of RCC with AML in the same kidney is rare, even more in patients without TS. Such cases should be accumulated and studied, to better understand the relationship among different types of renal tumors and to improve their management. In addition, this case is an opportunity to remind physicians to think of TS in front of this kind of tumor association which is most often seen in the context of this complex and to be aware of the various clinical and genetic diagnostic criteria to confirm or eliminate this diagnosis because the management will not be limited in these cases to the treatment of tumors.

## Figures and Tables

**Table 1:** Updated diagnostic criteria for tuberous sclerosis complex 2012 (reproduced from Northrup and Krueger 2013).

A. Genetic diagnostic criteria The identification of either a TSC1 or TSC2 pathogenic mutation in DNA from normal tissue is sufficient to make a definite diagnosis of TS. (note that 10% to 25% of TS patients have no mutation identified by conventional genetic testing, and a normal result does not exclude TS or has any effect on the use of clinical diagnostic criteria to diagnose TS).
B. Clinical diagnostic criteria Major features 1. Hypomelanotic macules (≥3, at least 5 mm diameter) 2. Angiofibromas (≥3) or fibrous cephalic plaque 3. Ungual fibromas (≥2) 4. Shagreen patch 5. Multiple retinal hamartomas 6. Cortical dysplasias 7. Subependymal nodules 8. Subependymal giant cell astrocytoma 9. Cardiac rhabdomyoma 10. Lymphangioleiomyomatosis^∗^ 11. Angiomyolipomas (≥2)^∗^ Minor features 1. “Confetti” skin lesions 2. Dental enamel pits (>3) 3. Intraoral fibromas (≥2) 4. Retinal achromic patch 5. Multiple renal cysts 6. Nonrenal hamartomas Definite diagnosis: two major features or one major feature with ≥2 minor features Possible diagnosis: either one major feature or ≥2 minor features ^∗^A combination of the two major clinical features (LAM and angiomyolipomas) without other features does not meet criteria for a definite diagnosis.

## Abbreviations

RCN: Renal cell neoplasm
AML: Angiomyolipomas
TS: Tuberous sclerosis
RCC: Renal cell carcinomas
CCRCC: Clear cell renal cell carcinoma
PEComas: Perivascular epithelioid cell tumors
CSCs: Cancer stem cells
CT: Computed tomography
MRI: Magnetic resonance imaging.
